# Photobiomodulation Therapy (PBMT) with Dual-Wavelength Enhances Reduction of Inflammation After Third Molar Extraction Compared with Red Laser PBMT: A Randomized Clinical Trial

**DOI:** 10.3390/jcm15072467

**Published:** 2026-03-24

**Authors:** Davisson Alves Pereira, Mariana Silva Bonatto, Carlos José Soares, Samara de Souza Santos, Roberto Sales e Pessoa, Maurício Andres Tinajero Aroni, Guilherme José Pimentel Lopes de Oliveira

**Affiliations:** 1 Department of Periodontology, School of Dentistry, Federal University of Uberlândia—UFU, Pará, Av., 1760-1844-Umuarama, Uberlândia 38405-320, Brazil; davisoncaneda@hotmail.com (D.A.P.); mariana.bonatto@hotmail.com (M.S.B.); carlosjsoares@ufu.br (C.J.S.); samarasmsantos@gmail.com (S.d.S.S.); 2 School of Dentistry, Universidade do Triangulo-UNITRI, Uberlândia 38411-849, Brazil; rp@inpes.com.br; 3 Department of Periodontology, School of Dentistry, Universidad Internacional del Ecuador (UIDE), Quito 170411, Ecuador; matinajeroar@uide.edu.ec

**Keywords:** bone, healing, photobiomodulation, soft tissue, third molar

## Abstract

**Background/Objectives:** Photobiomodulation (PBMT) has been shown to improve tissue healing; however, the best protocol for different clinical challenges is not clearly determined. Despite the good previous outcomes of the PBMT in healing of the third molar surgical sites, the ideal protocol of PBMT was not determined. The objective of this split-mouth double-blinded randomized clinical trial was to compare the effect of photobiomodulation (PBMT) with red and infrared wavelengths combined and PBMT with only red wavelength on the healing of post-extraction alveoli of third molars. **Methods:** Twenty patients underwent third molar extraction. The alveoli were treated randomly in a split mouth model with: PBMT with red laser (R-PBMT) or PBMT with red and infrared laser combined (IR-R-PBMT). PBMT was applied immediately, and 3 and 7 days after surgery. Patients were clinically evaluated in relation to repair (bleeding, exudate, color, and consistency of the tissues), degree of the edema, and through the application of a VAS scale (pain, edema, bleeding, chewing, and mouth opening) in the baseline period, and 3, 7, 14, 30, and 90 days after the surgical procedure. In addition, bone tissue density and structure were measured by radiographic analysis at 7 and 90 days postoperatively. **Results:** Clinical analysis showed that IR-R-PBMT induce more reduction in the edema 7 days after surgery compared with the R-PBMT; however, no significant differences were noted between groups in other parameters. **Conclusions:** IR-R-PBMT reduces the edema after 7 days of third molar extraction compared with the R-PBMT. Registration: This study was registered with the Brazilian Registry of Clinical Trials (REBEC-RBR-103g7j28; date of registration 12 July 2023) under number U1111-1297-6962.

## 1. Introduction

A range of surgical procedures are routinely performed in clinical dental practice [1,2,3,4,5,6], with great variations in techniques and approaches. However, in general, invasive procedures can cause patients a certain degree of postoperative discomfort [7]. Postoperative morbidity is related to inflammatory processes, which are the result of surgical interventions that lead to the local release of biological mediators of inflammation (i.e., pro-inflammatory cytokines, arachidonic acid metabolites, bradykinin, and histamine) [8,9]. One way of managing wounds resulting from surgical procedures in the oral cavity is the use of low-intensity laser photobiomodulation (PBMT) [10]. PBMT has shown promising results in reducing painful symptoms associated with temporomandibular disorders [11], as well as in muscle lesions [12], and after surgical procedures [13,14]. In addition, PBMT has been shown to improve the repair process in grafted and non-grafted bone defects [15], in neural lesions [16], and in soft tissues lesions [17].

However, factors inherent to the physical properties of lasers (i.e., irradiation time, power, energy density, and mode of application) can influence the success of PBMT [15,17]. Among these factors, the wavelength has been highlighted, as it presents different properties in relation to tissue penetrability, even in cases with the application of a similar amount of energy and number of therapy sessions [18,19]. Lasers with wavelengths within the infrared light range have greater tissue penetrability, which makes them preferable when a deep tissue approach is indicated (i.e., osseointegration of implants, treatment of paresthesias) [18], while lasers that emit light within the red wavelength range have less tissue penetration, but there is a concentration of energy in the most superficial regions of the wound, which makes this type of PBMT more suitable for superficial tissue approaches (i.e., treatment of mucositis) [17].

Our research group has proposed a way of simplifying the therapeutic protocol. PBMT can be applied with the association of lasers with red and infrared wavelengths simultaneously and combined, which could be beneficial in situations where there is a need to stimulate both superficial and deep tissue repair [20,21]. An example of a clinical condition that could benefit from this type of approach is the post-extraction socket maneuver after third molar extraction, as this type of surgery may involve the need for extensive tissue detachment and wear of surrounding bone tissue, generating a considerable inflammatory process [22]. In addition, the defects resulting from the tooth extraction have superficial and deep components, and distinct wavelengths may impact the entire area of the post-extraction sockets during the healing phase [20,21,22].

Due to the varied therapeutic possibilities of PBMT, it is necessary to improve outcomes and obtain more predictable results by establishing more precise protocols and indications for this treatment modality. For this purpose, the objective of this split-mouth randomized, controlled trial is to evaluate the effect of PBMT in third molar extraction sockets using the red wavelength or with dual-wavelength (red and infrared). The null hypothesis of this study was that these different protocols would have similar clinical results during the postoperative course of third molar extractions.

## 2. Materials and Methods

### 2.1. Study Design

This split-mouth randomized, controlled trial was carried out following the CONSORT protocol. The sample consisted of 20 patients (11 females and 9 males) who were approached at the clinic of Federal University of Uberlândia. These patients underwent procedures for dental extraction of four third molars and a subsequent stimulation procedure for healing of the post-extraction alveoli with PBMT with infra-red and red laser (IR-R-PBMT) on one side twice, and on the contralateral side 2 further irradiations with red laser (R-PBMT) were performed. The randomization process to select treatments in each well was based on the use of an online randomization table (random.org) (GJO). The researcher responsible for the treatment allocation does not participate in the surgeries and evaluations. The patients were blinded regarding the protocols of PBMT applied. This study was conducted in accordance with the Declaration of Helsinki and was approved for conduction by the Human Research Ethics Committee of the Federal University of Uberlândia under protocol number CAAE: 72834023.3.0000.5152, and it was registered with the Brazilian Registry of Clinical Trials (REBEC-RBR-103g7j28-Date of registration 7 December 2023) under number U1111-1297-6962. All patients read and signed an informed consent form before the enrolment in this study.

### 2.2. Sample Size Calculation

The sample calculation was performed based on the study by Heo et al., 2002 [23], which carried out fractal analysis of bone in the regeneration phase after orthognathic surgery. A fixed mean difference of 0.10 points was established as being clinically relevant, and with an expected mean standard deviation of 0.05, a sample size of 15 patients was calculated to be sufficient to determine this level of difference, with a β power of 0.90 and an α power of 0.05. In this way, 20 patients were treated per group to replace any patients who withdrew during the project.

### 2.3. Inclusion and Exclusion Criteria

To be included in this study, patients were required to present the following characteristics: be over 18 years of age, have good oral hygiene, have four third molars, and be indicated for tooth extraction of upper and lower third molars. Patients who presented periodontal disease or systemic diseases or conditions that alter the bone turnover were excluded.

### 2.4. Surgical Procedure

The third molars were classified according to the Pell and Gregory and Winter classification systems. The classification of tooth position was important to determine whether the level of surgical difficulty and invasiveness of third molar extraction was comparable between the experimental groups. On the day of surgery, all patients received 8 mg of dexamethasone orally one hour before the procedure. Local anesthesia was administered, and when necessary, the teeth were accessed through a full-thickness flap and extracted using appropriate surgical instruments. All four third molars were extracted on the same day. The surgical sites were sutured with simple interrupted stitches using 5-0 nylon sutures (Ethicon^®^, Johnson & Johnson, São Paulo, Brazil). The postoperative medication protocol for all patients consisted of diclofenac sodium (50 mg) every 8 h for 3 days and dipyrone sodium (500 mg) every 6 h for 3 days, both administered orally. Patients were instructed to maintain oral hygiene; however, no standardization of toothbrushes or toothpaste was implemented. A liquid and soft diet was recommended for the first 3 postoperative days. Additionally, a 0.12% chlorhexidine digluconate mouthwash was prescribed for 7 consecutive days to be used every 12 h. The sutures were removed 7 days after the surgical procedure. The surgical procedures were executed by two experienced operators (DAP and MSB).

### 2.5. Photobiomodulation Protocol

The InAlAs/GaAlAs laser device (XTherapy EC; λ = 660 nm and λ = 808 nm; 100 mW; ϕ ≈ 0.600 µm; tip divergence = 0.37 rad; continuous wave; spot area = 0.0283 cm^2^; DMC Equipamentos, São Carlos, SP, Brazil) was used to perform the irradiations. Laser irradiation was performed immediately after the extractions and suturing procedures, and on the 3rd and 7th postoperative days. On the side treated with IR-R-PBMT applied simultaneously and in combination, two irradiations were performed per alveolus, with 4 J delivered per irradiation, totaling 8 J per alveolus and 16 J per treated side per session. On the side treated with R-PBMT, the irradiation protocol followed the same parameters as described above, with the only difference being the emitted wavelength; therefore, 8 J of energy was delivered per application in each alveolus. The irradiation energy density used per session was approximately 859.92 J/cm^2^ per session and 2579.76 J/cm^2^ throughout the treatment for each protocol. The PBMT irradiation time was 40 s in the IR-R-PBMT group and 80 s in the R-PBMT group, using scanning mode.

### 2.6. Clinical Analysis

Patients were clinically evaluated 3, 7, 14, 30, and 90 days after the surgical procedure. For patient-centered qualitative analyses, a VAS scale graduated from 0 to 10 was applied, with 0 considered the best result, where patients were asked about their perceptions regarding edema, pain, bleeding, opening of the mouth, and limitations on the chewing. Wound healing assessment was based on a combination of clinical parameters related to tissue inflammatory status and healing quality, including bleeding, tissue color, exudate, and tissue consistency. These parameters were evaluated according to a previously described scoring system [14], generating an overall healing score ranging from 4 to 12, with lower scores indicating better healing conditions: (I) Bleeding: (1) No bleeding, (2) Induced by palpation, (3) Spontaneous; (II) Tissue color: (1) 100% pink gum; (2) <50% of hyperemic and mobile gingiva; (3) >50% of hyperemic and mobile gingiva; (III) Exudate: (1) Absent, (2) Present associated with the presence of pronounced plaque around the alveolus, (3) Suppuration; (IV) Consistency of the healing tissue: (1) Resilient and pink, (2) Flabby and red, (3) Fragile and green or grayish. Regarding quantitative analyses, mouth opening assessments were carried out by measuring the interincisal distance at the midline with the patient at maximum mouth opening in a comfortable position. In addition, vertical and horizontal dimensions of the face were measured with the aim of establishing post-surgical edema: vertical measurement: lateral extremity of the external palpebral commissure to the angle of the mandible; horizontal measurement: ear tragus to ipsilateral external labial commissure [14]. The results of measurements of facial distances and mouth opening were expressed as the variation in values analyzed in the experimental period compared to measurements performed before the surgical procedure. All the clinical analyses were performed by a single, calibrated and blinded evaluator (SSS).

### 2.7. Panoramic X-Ray Analysis

Panoramic radiographs were obtained at 7 and 90 days after the surgical procedure using the Eagle Edge panoramic unit (Dabi Atlante, Ribeirão Preto, SP, Brazil). All images were acquired under the manufacturer’s automatic exposure mode for adult patients, in which tube voltage (kVp), tube current (mA), and exposure time are automatically adjusted according to the patient’s anatomical characteristics. Both time-point examinations were performed using the same device and identical automatic acquisition protocol, with no manual modification of exposure parameters, ensuring methodological standardization and minimizing variability related to energy settings. To perform the density analysis, ImageJ software (version 1.3; National Institutes of Health, Bethesda, MD, USA) was used, with the “histometry” tool in a region of interest (ROI) of 32 × 32 pixels in the center of each alveolus, making it possible to obtain the values of gray tones present in the X-ray and to compare the two moments. Alveolar density and dimension fractal analyses were performed for each alveolus. After 30 days, thirty percent of the samples were randomly selected and reanalyzed by the same examiner to assess intra-examiner reproducibility. Reliability was evaluated using the intraclass correlation coefficient (ICC), demonstrating excellent agreement (ICC = 0.93, 95% CI: 0.88–0.95). All radiographic analyses were performed by a single calibrated and blinded examiner (ECS).

### 2.8. Statistical Analysis

The distribution of third molars according to Pell and Gregory and Winter classifications was compared between groups using Fisher’s exact test. The effects of the treatment (IR-R-PBMT vs. R-PBMT), evaluation period, and tooth location (upper vs. lower) on facial dimensions and VAS scores were analyzed using linear mixed-effect models. In these models, treatment, period, and location were included as fixed factors, while subject identity was included as a random factor to account for repeated measures within individuals. Interaction effects between treatment and period were also evaluated. Model assumptions of normality and homogeneity of variance were checked using residual plots. Statistical analyses were performed using Jamovi software (Version 2.6.44), and a significance level of 5% was adopted for all tests.

## 3. Results

### 3.1. Level of Difficulty of the Extraction Procedure and the Occurrence of Complications Were Similar Between the Different PBMT Protocols

**Figure 1:**
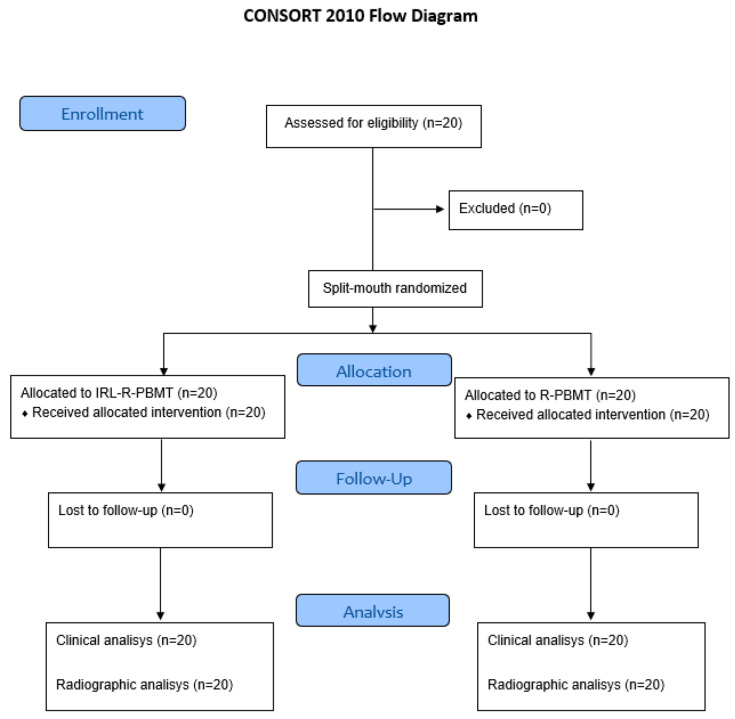
Flow diagram of the study.

The patients were treated and follow-up was conducted between July to November 2023. There were no patient withdrawals from this study (Figure 1). A total of 80 teeth were extracted from the 20 patients in this study, 40 lower teeth and 40 upper teeth, half of which were treated with IR-R-PBMT and the other half with R-PBMT. In total, 24 osteotomies (one upper and 12 lower teeth whose alveoli were treated by R-PBMT and 11 lower teeth whose alveoli were treated by IR-R-PBMT) and 17 odontosections (eight lower teeth treated by R-PBMT and nine lower teeth treated by IR-R-PBMT) were performed during the dental extraction surgical procedure, with no statistically significant differences between the groups. The average surgery time was 60.06 ± 25.85 min, and there were no differences in relation to the positioning of the teeth between the experimental groups according to Winter’s (Table 1) and Pell and Gregory’s classifications (Table 1). There were also no differences between the groups in relation to the frequency of unpleasant repercussions related to surgical procedures. The main negative repercussions were trismus (one case at 3 days and three cases at 7 days), the presence of bone spicules within a 7-day period in a patient associated with the alveoli of both upper molars, and the need for irrigation with chlorhexidine and saline solution in two lower molar alveoli in one patient within a 7-day period.

All surgical procedures were performed under local anesthesia following a standardized surgical protocol. The choice of surgical approach was determined by tooth position and impaction characteristics, according to Winter’s and Pell and Gregory’s classifications. In cases requiring osteotomy and/or odontosection, a full-thickness mucoperiosteal flap was elevated to allow adequate visualization and bone removal. In contrast, in cases where tooth extraction could be accomplished without the need for bone removal or tooth sectioning, a flapless approach was adopted. Although two experienced surgeons performed the procedures, all surgeries followed the same standardized protocol to ensure consistency across cases. There were no statistically significant differences between the experimental groups regarding surgical difficulty variables (osteotomy, odontosection, operative time, and tooth position classification).

### 3.2. Degree of Healing and Symptoms Noted During the Postoperative Phase of Third Molar Extraction by Professionals and Patients Were Not Different Between the Different PBMT Protocols

The primary clinical outcomes assessed in this study included pain intensity, measured using a visual analog scale (VAS), postoperative edema, trismus, and soft tissue healing. Pain scores were evaluated at 1, 3, 7, and 14 days after surgery, showing a gradual reduction over time in both experimental groups, with no statistically significant differences between R-PBMT and IR-R-PBMT treatments. Edema and trismus were evaluated using standardized clinical criteria and similarly demonstrated progressive improvement, with most cases resolving by the 7th postoperative day. Soft tissue healing was assessed at each follow-up visit, revealing satisfactory mucosal closure in all patients and no significant differences between groups. Detailed values and statistical comparisons are provided in Table 2.

### 3.3. There Was a Reduction in the Inflammatory Process in Both Groups, However, the IR-R-PBMT Protocol Was Associated with Greater Reductions in Edema over a 7-Day Period than the R-PBMT Protocol

Following tooth extraction, a decrease in mouth opening and an increase in bilateral facial dimensions were observed, with the most pronounced changes occurring between 3 and 7 days postoperatively. Thereafter, mouth opening progressively improved, and facial dimensions returned toward baseline values. Across the 7-day follow-up, the IR-R-PBMT group exhibited smaller variations in both horizontal and vertical facial dimensions compared to the R-PBMT group, suggesting that the IR-R-PBMT protocol was associated with reduced postoperative edema (Table 3).

### 3.4. IR-R-PBMT and R-PBMT Showed Similar Potential for Bone Tissue Healing

**Figure 2:**
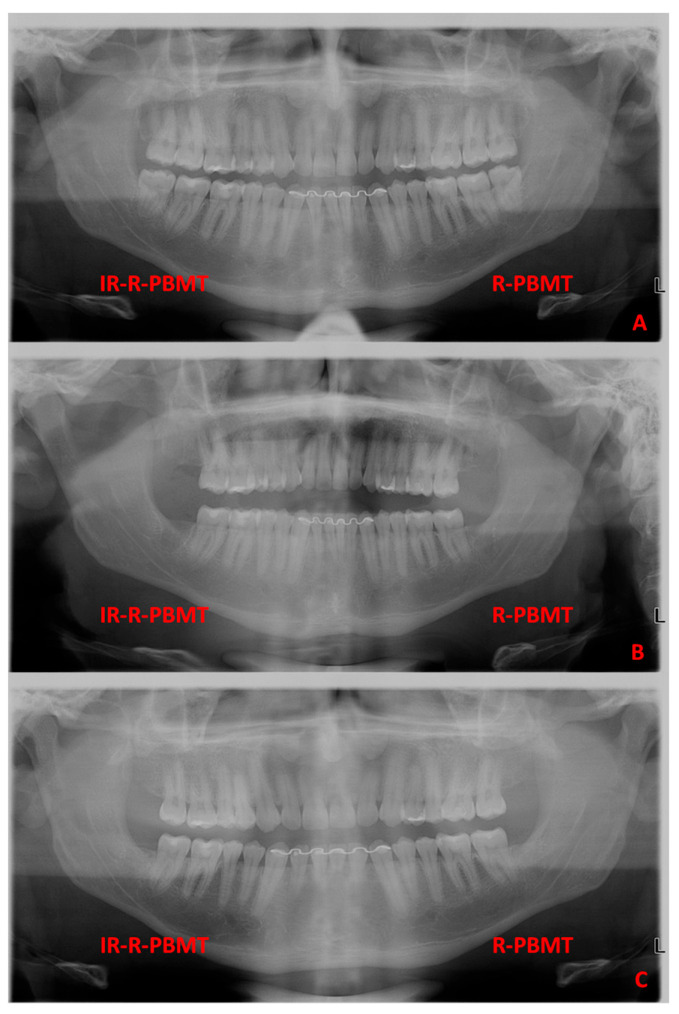
Representative panoramic radiographic images of a patient enrolled in this study. (**A**) Pre-surgical condition of the third molars; (**B**) radiographical aspect of the extraction sockets after 7 days of the surgical procedure; (**C**) the radiographical aspect of the extraction sockets after 90 days of the surgical procedure. It was not possible to detect differences in radiographical density and fractal dimension in the post-extraction sockets treated with IR-R-PBMT and R-PBMT.

No differences were observed between the groups in relation to bone density and fractal dimension; however, in both groups there was an increase in bone density in the 90-day period compared to the baseline period (*p* < 0.05) (Figure 2) (Table 4).

## 4. Discussion

The IR-R-PBMT protocol used in this study modulates the postoperative clinical course after third molar surgery, as it reduced edema over a 7-day period; however, this reduction was considered clinically negligible. No other differences in postoperative outcomes were observed between the IR-R-PBMT and R-PBMT groups. Therefore, the alternative hypothesis of this study was only partially supported.

This reduction in edema promoted by IR-R-PBMT corroborates data from a previous study that observed a reduction in edema and pain and improvement in soft tissue repair after extraction of lower third molars compared to sockets that were not subjected to PBMT [24]. Although this study did not include a control group without PBMT, the results suggest that R-PBMT may also enhance tissue healing. The addition of the infrared wavelength could have further contributed to edema reduction by reaching deeper tissues. However, the decrease in swelling in IR-R-PBMT compared with R-PBMT was minimal, as evidenced by the absence of a significant effect on mouth opening, which serves as an indirect indicator of facial edema. The anti-inflammatory effects observed with IR-R-PBMT may be due to the modulatory effects of this therapy observed in cell culture studies. The infrared PBMT accelerated the proliferation and migration of fibroblasts and increased their ability to synthesize collagen and produce angiogenic factors even when these cells were challenged with pro-inflammatory cytokines (TNF-α, IL-1β, IL-6, and IL-8) [25]. In a study with macrophage cultures, it was observed that infrared PBMT accelerated the differentiation of undifferentiated mesenchymal cells into macrophages and increased the conversion of M1 to M2 macrophages; this event was associated with a reduction in pro-inflammatory cytokines TNFα and IL6 [26]. A preclinical study demonstrated that infrared PBMT reduced edema formation in the paws of mice that were injected with phlogistic agents [27]. Anti-inflammatory effects have also been reported with the use of red laser, including the regulation of key signaling pathways involved in macrophage polarization (PI3K/AKT/mTOR, NF-κB, and STAT) [28], as well as the reduction in pro-inflammatory cytokine expression in fibroblast and osteoblast cultures [29].

The mechanism of action of PBMT with red and infrared light is similar and targets the action of light energy on cellular mitochondrial chromophores, accelerating metabolism by stimulating the process of cellular respiration [30]. In this way, red and infrared laser can stimulate the proliferation, migration, differentiation, and action of different cell types [31,32]. The difference between red and infrared lasers is due to their tissue penetrability power, as the red laser is more absorbed superficially, while the infrared laser has greater penetrability power [28,33]. Post-extraction alveoli are clinical conditions that can benefit from the interaction of PBMT with the red and infrared laser since these types of lesions have superficial and deep components that can be affected differently by red and infrared light [22]. Interestingly, the clinical analyses of superficial tissues were not different between the groups, while differences between the protocols tested in this study were observed in the analysis of edema, which included the assessment of deep tissues. Therefore, the findings of this study are in agreement with the applicability of red light in the healing of superficial tissues and of infrared light in deep tissues [33].

In the current study, patients noticed no differences between the treated and untreated sides. This absence may have occurred due to the study design using a split-mouth model, which makes it difficult for the patient to distinguish differences between therapies, as well as the fact that the procedure was performed in a single surgical procedure by experienced professionals, which may have reduced the postoperative morbidity. However, it is important to note that no differences were observed between IR-R-PBMT and R-PBMT in other parameters, such as chewing discomfort, edema, or difficulty in mouth opening. The healing process of soft tissues was also similar. Indeed, both red and infrared lasers have previously been shown to influence the formation of extracellular matrix products and growth factors in in vitro studies [26,29], as well as to demonstrate potential in wound healing in the dorsal regions of rabbits [34] and rats [35]. The fact that it was not possible to observe postoperative clinical differences related to the level of surgical complexity and the different protocols used seems to make it clear that the postoperative repercussions are not related to the position of the teeth and the protocols used. It is possible that in more difficult surgical conditions, which require longer procedure execution times, IR-R-PBMT may prove superior in controlling postoperative morbidity and may be better distinguished by patients. This hypothesis should be tested in the future.

There were no differences between the PBMT protocols regarding the formation and structure of bone tissue in the post-extraction sockets. These findings may have occurred because both protocols act in a similar way on bone tissue. However, it is worth noting that studies that demonstrate the effects of PBMT on bone formation present a greater number of PBMT sessions than the protocols tested in the current study. A previous clinical study did not demonstrate differences in the tomographic appearance of post-extraction alveoli of third molars treated or not with IR-R-PBMT [26]; therefore, the protocol tested in this study is not realistically intended to accelerate bone formation in post-extraction sockets.

The current study presents some limitations that should be considered when interpreting the results. The relatively small sample size may limit the generalizability of the findings. Although the split-mouth design effectively minimizes inter-individual variability, performing the surgical procedures on the same day could have introduced confusion in patient-reported outcomes, particularly regarding VAS pain scores. Additionally, two different operators performed the surgeries, which may have contributed to operator-related variability despite adherence to a standardized protocol. Patient-related behaviors and compliance with postoperative care could also represent potential sources of bias. It is possible that the standardized medications administered before and after surgery (including dexamethasone and diclofenac sodium) may have influenced or partially masked differences in postoperative pain perception between groups. Although the pharmacological protocol was identical for all participants, the actual consumption of analgesics was not individually recorded, preventing correlation analysis between medication intake and VAS pain scores. This should be considered a limitation of the present study. Future clinical trials should include strict monitoring of postoperative analgesic consumption to allow more accurate interpretation of pain-related outcomes. Evaluating the PBMT protocol under more complex clinical scenarios could help identify patients who would truly benefit from this therapy and its different variations, as well as assess whether the future use of anti-inflammatory and analgesic drugs could be reduced if PBMT proves sufficiently effective in consistently controlling inflammation and the pain associated with third molar extraction.

## 5. Conclusions

It can be concluded that IR-R-PBMT was associated with a reduction in postoperative edema at 7 days compared with R-PBMT, although the clinical impact of this difference was modest. No significant differences were observed between the IR-R-PBMT and R-PBMT groups regarding soft tissue healing or bone formation. Based on these findings, the null hypothesis was partially rejected, since a difference was observed only for postoperative edema, while the other evaluated outcomes showed no significant differences between the groups.

## Figures and Tables

**Table 1:** Classification of tooth positioning according to Winter and Pell and Gregory.

Classification/Group	IR-R-PBMT		R-PBMT	
Winter Classification	Upper	Lower	Upper	Lower
Vertical	18	14	16	11
Disto-angled	1	0	4	0
Mesio-angulated	1	5	0	4
Horizontal	0	1	0	5
Total	20	20	20	20
Pell and Gregory Classification				
A	13	14	13	10
B	3	2	6	8
C	7	4	1	2
Total	20	20	20	20

**Table 2:** Median and interquartile range values of healing index, VAS scale for pain, edema, bleeding, mouth opening, and chewing.

Parameter	Period/Group	IR-R-PBMT	R-PBMT
Healing index upper	3 days	5.50 (5.00–7.00)	5.00 (4.00–7.00)
	7 days	6.00 (4.75–7.00)	6.00 (4.00–7.00)
	15 days	4.00 (4.00–5.00)	4.00 (4.00–4.25)
	30 days	4.00 (4.00–5.00)	4.00 (4.00–4.00)
	90 days	4.00 (4.00–4.00)	4.00 (4.00–4.00)
Healing index lower	3 days	5.50 (5.00–6.00)	5.00 (4.75–6.25)
	7 days	6.00 (4.75–7.00)	5.00 (4.00–7.00)
	15 days	5.00 (4.00–5.00)	5.00 (4.00–5.00)
	30 days	4.00 (4.00–5.00)	4.00 (4.00–4.25)
	90 days	4.00 (4.00–4.00)	4.00 (4.00–4.00)
Pain	3 days	3.00 (1.00–6.00)	3.00 (1.00–5.00)
	7 days	2.00 (0.00–6.50)	2.50 (0.00–7.00)
	15 days	0.00 (0.00–2.00)	0.00 (0.00–1.25)
	30 days	0.00 (0.00–0.00)	0.00 (0.00–0.00)
	90 days	0.00 (0.00–0.00)	0.00 (0.00–0.00)
Edema	3 days	5.00 (1.50–8.00)	3.00 (1.50–8.00)
	7 days	2.00 (0.00–5.75)	2.00 (0.00–5.00)
	15 days	0.00 (0.00–1.25)	0.00 (0.00–2.00)
	30 days	0.00 (0.00–0.00)	0.00 (0.00–0.00)
	90 days	0.00 (0.00–0.00)	0.00 (0.00–0.00)
Bleeding	3 days	2.00 (0.00–5.00)	2.00 (0.00–6.00)
	7 days	1.00 (0.00–2.00)	0.50 (0.00–2.00)
	15 days	0.00 (0.00–0.00)	0.00 (0.00–0.00)
	30 days	0.00 (0.00–0.00)	0.00 (0.00–0.00)
	90 days	0.00 (0.00–0.00)	0.00 (0.00–0.00)
Chewing	3 days	7.00 (3.50–9.00)	5.00 (3.50–8.00)
	7 days	4.00 (1.00–6.25)	4.00 (1.75–7.00)
	15 days	1.00 (0.00–1.25)	0.50 (0.00–2.00)
	30 days	0.00 (0.00–0.00)	0.00 (0.00–0.00)
	90 days	0.00 (0.00–0.00)	0.00 (0.00–0.00)
Mouth opening	3 days	5.00 (4.50–8.00)	6.00 (4.50–8.00)
	7 days	2.50 (0.75–7.00)	2.50 (0.00–5.25)
	15 days	0.00 (0.00–1.25)	0.00 (0.00–1.25)
	30 days	0.00 (0.00–0.00)	0.00 (0.00–0.00)
	90 days	0.00 (0.00–0.00)	0.00 (0.00–0.00)

**Table 3:** Mean and standard deviation values for variation in facial dimensions and mouth opening. * *p* < 0.05—lower values of facial dimension variation than observed in the R-PBMT group—linear mixed models.

Parameter	Period/Group	IR-R-PBMT	R-PBMT
Mouth opening	Baseline	40.91 ± 8.36	
	ΔBas–3d	17.74 ± 8.23	
	ΔBas–7d	9.36 ± 8.00	
	ΔBas–15d	2.77 ± 7.94	
	ΔBas–30d	−0.79 ± 6.79	
	ΔBas–90d	−3.36 ± 6.42	
Vertical dimension	Baseline	10.97 ± 0.93	10.86 ± 0.76
	ΔBas–3d	0.23 ± 1.19	0.28 ± 1.08
	ΔBas–7d	−0.32 ± 1.10 *	0.31 ± 0.97
	ΔBas–15d	−0.16 ± 1.05	−0.01± 0.88
	ΔBas–30d	−0.18 ± 0.86	−0.02 ± 0.87
	ΔBas–90d	−0.07 ± 0.67	0.05 ± 0.66
Horizontal dimension	Baseline	11.69 ± 0.89	11.68 ± 0.86
	ΔBas–3d	0.15 ± 0.70	0.14 ± 0.63
	ΔBas–7d	−0.27 ± 0.69 *	0.26 ± 0.60
	ΔBas–15d	−0.05 ± 0.66	0.00 ± 0.59
	ΔBas–30d	−0.13 ± 0.53	−0.06 ± 0.49
	ΔBas–90d	0.03 ± 0.67	−0.17 ± 0.62

**Table 4:** Mean and standard deviation values of radiographic density and fractal dimension through radiographic analysis.

Position	Parameter	Period/Group	IR-R-PBMT	R-PBMT
Upper	Radiographic density	Baseline	106.1 ± 20.66	105.9 ± 19.34
		90 days	134.6 ± 20.12	132.6 ± 19.12
	Fractal dimension	Baseline	1.21 ± 0.11	1.25 ± 0.09
		90 days	1.29 ± 0.14	1.28 ± 0.13
Lower	Radiographic density	Baseline	119.5 ± 15.70	117.1 ± 16.99
		90 days	131.3 ± 18.06	128.1 ± 18.67
	Fractal dimension	Baseline	1.30 ± 0.10	1.30 ± 0.13
		90 days	1.26 ± 0.16	1.30 ± 0.09

## Data Availability

The datasets generated and/or analyzed during the current study are available from corresponding author on request. The data will be included in the repository of the Universidade Federal de Uberlândia after pHD thesis finalization of Davisson Alves Pereira.

## Abbreviations

The following abbreviations are used in this manuscript:

PBMT	Photobiomodulation therapy
VAS	Visual analogic scale
ROI	Region of interesting
TNF	Tumor necrosis factor
IL	Interleukin
